# Supplementary material for: A UMLS-based spell checker for natural language processing in vaccine safety
Source: BMC Med Inform Decis Mak. 2007 Feb 12;7:3. doi: 10.1186/1472-6947-7-3 (PMC1805499; doi:10.1186/1472-6947-7-3)
Supplement: Additional file 13 — RAP application directory. Sets up directory for RAP (RDF application for PHP) source code [file 1472-6947-7-3-S13.gz › rap/doc/phpdoc/__filesource/fsource_default__vocabularyFOAF.php.html]

xml version="1.0" encoding="iso-8859-1"?


File Source for FOAF.php


# Source for file FOAF.php

Documentation is available at FOAF.php

```
1. <?PHP
2. // ----------------------------------------------------------------------------------
3. // Friend of a Friend (FOAF) Vocabulary
4. // ----------------------------------------------------------------------------------
5. // Version                   : 0.9
6. // Authors                   : Tobias Gau� (tobias.gauss@web.de)
7. //
8. // Description               : Wrapper, defining resources for all terms of the
9. //                               Friend of a Friend project (FOAF).
10. //                               For details about FOAF see: http://xmlns.com/foaf/0.1/.
11. //                                Using the wrapper allows you to define all aspects of
12. //                             the vocabulary in one spot, simplifing implementation and
13. //                             maintainence. Working with the vocabulary, you should use
14. //                             these resources as shortcuts in your code.
15. //
16. // ----------------------------------------------------------------------------------
17. // History:
18. // 11-08-2003                 : Initial version
19. // ----------------------------------------------------------------------------------


23. // FOAF concepts

25. $FOAF_Agent = new Resource(FOAF_NS . 'Agent');
26. $FOAF_Document = new Resource(FOAF_NS . 'Document');
27. $FOAF_Group = new Resource(FOAF_NS . 'Group');
28. $FOAF_Image = new Resource(FOAF_NS . 'Image');
29. $FOAF_OnlineAccount = new Resource(FOAF_NS . 'OnlineAccount');
30. $FOAF_OnlineChatAccount = new Resource(FOAF_NS . 'OnlineChatAccount');
31. $FOAF_OnlineEcommerceAccount = new Resource(FOAF_NS . 'OnlineEcommerceAccount');
32. $FOAF_OnlineGamingAccount = new Resource(FOAF_NS . 'OnlineGamingAccount');
33. $FOAF_Organization = new Resource(FOAF_NS . 'Organization');
34. $FOAF_Person = new Resource(FOAF_NS . 'Person');
35. $FOAF_PersonalProfileDocument = new Resource(FOAF_NS . 'PersonalProfileDocument');
36. $FOAF_Project = new Resource(FOAF_NS . 'Project');
37. $FOAF_accountName = new Resource(FOAF_NS . 'accountName');
38. $FOAF_accountServiceHomepage = new Resource(FOAF_NS . 'accountServiceHomepage');
39. $FOAF_aimChatID = new Resource(FOAF_NS . 'aimChatID');
40. $FOAF_based_near = new Resource(FOAF_NS . 'based_near');
41. $FOAF_currentProject = new Resource(FOAF_NS . 'currentProject');
42. $FOAF_depiction = new Resource(FOAF_NS . 'depiction');
43. $FOAF_depicts = new Resource(FOAF_NS . 'depicts');
44. $FOAF_dnaChecksum = new Resource(FOAF_NS . 'dnaChecksum');
45. $FOAF_family_name = new Resource(FOAF_NS . 'family_name');
46. $FOAF_firstName = new Resource(FOAF_NS . 'firstName');
47. $FOAF_fundedBy = new Resource(FOAF_NS . 'fundedBy');
48. $FAOF_geekcode = new Resource(FOAF_NS . 'geekcode');
49. $FOAF_gender = new Resource(FOAF_NS . 'gender');
50. $FOAF_givenname = new Resource(FOAF_NS . 'givenname');
51. $FOAF_holdsAccount = new Resource(FOAF_NS . 'holdsAccount');
52. $FOAF_homepage = new Resource(FOAF_NS . 'homepage');
53. $FOAF_icqChatID = new Resource(FOAF_NS . 'icqChatID');
54. $FOAF_img = new Resource(FOAF_NS . 'img');
55. $FOAF_interest = new Resource(FOAF_NS . 'interest');
56. $FOAF_jabberID = new Resource(FOAF_NS . 'jabberID');
57. $FOAF_knows = new Resource(FOAF_NS . 'knows');
58. $FOAF_logo = new Resource(FOAF_NS . 'logo');
59. $FOAF_made = new Resource(FOAF_NS . 'made');
60. $FOAF_maker = new Resource(FOAF_NS . 'maker');
61. $FOAF_mbox = new Resource(FOAF_NS . 'mbox');
62. $FOAF_mbox_sha1sum = new Resource(FOAF_NS . 'mbox_sha1sum');
63. $FOAF_member = new Resource(FOAF_NS . 'member');
64. $FOAF_membershipClass =new Resource(FOAF_NS . 'membershipClass');
65. $FOAF_msnChatID = new Resource(FOAF_NS . 'msnChatID');
66. $FOAF_myersBriggs = new Resource(FOAF_NS . 'myersBriggs');
67. $FOAF_name = new Resource(FOAF_NS . 'name');
68. $FOAF_nick = new Resource(FOAF_NS . 'nick');
69. $FOAF_page = new Resource(FOAF_NS . 'page');
70. $FOAF_pastProject = new Resource(FOAF_NS . 'pastProject');
71. $FOAF_phone = new Resource(FOAF_NS . 'phone');
72. $FOAF_plan = new Resource(FOAF_NS . 'plan');
73. $FOAF_primaryTopic = new Resource(FOAF_NS . 'primaryTopic');
74. $FOAF_publications = new Resource(FOAF_NS . 'publications');
75. $FOAF_schoolHomepage = new Resource (FOAF_NS . 'schoolHomepage');
76. $FOAF_sha1 = new Resource (FOAF_NS . 'sha1');
77. $FOAF_surname = new Resource (FOAF_NS . 'surname');
78. $FOAF_theme = new Resource(FOAF_NS . 'theme');
79. $FOAF_thumbnail = new Resource(FOAF_NS . 'thumbnail');
80. $FOAF_tipjar = new Resource(FOAF_NS . 'tipjar');
81. $FOAF_title = new Resource(FOAF_NS . 'title');
82. $FOAF_topic = new Resource(FOAF_NS . 'topic');
83. $FOAF_topic_interest = new Resource(FOAF_NS . 'topic_interest');
84. $FOAF_weblog = new Resource(FOAF_NS . 'weblog');
85. $FOAF_workInfoHomepage = new Resource(FOAF_NS . 'workInfoHomepage');
86. $FOAF_workplaceHomepage = new Resource(FOAF_NS . 'workplaceHomepage');
87. $FOAF_yahooChatID = new Resource(FOAF_NS . 'yahooChatID');


92. ?>
```

Documentation generated on Fri, 17 Dec 2004 16:14:51 +0100 by phpDocumentor 1.3.0RC3
